# Expression profiling of cell-intrinsic regulators in the process of differentiation of human iPSCs into retinal lineages

**DOI:** 10.1186/s13287-018-0848-7

**Published:** 2018-05-11

**Authors:** Jen-Hua Chuang, Aliaksandr A. Yarmishyn, De-Kuang Hwang, Chih-Chien Hsu, Mong-Lien Wang, Yi-Ping Yang, Ke-Hung Chien, Shih-Hwa Chiou, Chi-Hsien Peng, Shih-Jen Chen

**Affiliations:** 1 Department of Medical Research, Taipei Veterans General Hospital, Taipei, Taiwan; 2 Department of Ophthalmology, Taipei Veterans General Hospital, Taipei, Taiwan; 3 Institute of Pharmacology, National Yang-Ming University, Taipei, Taiwan; 4 Department of Ophthalmology, Tri-Service General Hospital & National Defense Medical Center, Taipei, Taiwan; 5 School of Medicine, National Yang-Ming University, Taipei, Taiwan; 6 Department of Ophthalmology, Shin Kong Wu Ho-Su Memorial Hospital and Fu Jen Catholic University, Taipei, Taiwan

**Keywords:** Human induced pluripotent stem cells, Retina, Retinal ganglion cells, Optic vesicles, Retinal pigment epithelium, DNA microarrays, Collier/Olf1/EBF, EBF1

## Abstract

**Background:**

Differentiation of human induced pluripotent stem cells (hiPSCs) into retinal lineages offers great potential for medical application. Therefore, it is of crucial importance to know the key intrinsic regulators of differentiation and the specific biomarker signatures of cell lineages.

**Methods:**

In this study, we used microarrays to analyze transcriptomes of terminally differentiated retinal ganglion cell (RGC) and retinal pigment epithelium (RPE) lineages, as well as intermediate retinal progenitor cells of optic vesicles (OVs) derived from hiPSCs. In our analysis, we specifically focused on the classes of transcripts that encode intrinsic regulators of gene expression: the transcription factors (TFs) and epigenetic chromatin state regulators. We applied two criteria for the selection of potentially important regulators and markers: firstly, the magnitude of fold-change of upregulation; secondly, the contrasted pattern of differential expression between OV, RGC and RPE lineages.

**Results:**

We found that among the most highly overexpressed TF-encoding genes in the OV/RGC lineage were three members of the Collier/Olfactory-1/Early B-cell family: *EBF1*, *EBF2* and *EBF3*. Knockdown of *EBF1* led to significant impairment of differentiation of hiPSCs into RGCs. EBF1 was shown to act upstream of ISL1 and BRN3A, the well-characterized regulators of RGC lineage specification. TF-encoding genes *DLX1*, *DLX2* and *INSM1* were the most highly overexpressed genes in the OVs, indicating their important role in the early stages of retinal differentiation. Along with *MITF*, the two paralogs, *BHLHE41* and *BHLHE40*, were the most robust TF markers of RPE cells. The markedly contrasted expression of *ACTL6B,* encoding the component of chromatin remodeling complex SWI/SNF, discriminated hiPSC-derived OV/RGC and RPE lineages.

**Conclusions:**

We identified novel, potentially important intrinsic regulators of RGC and RPE cell lineage specification in the process of differentiation from hiPSCs. We demonstrated the crucial role played by EBF1 in differentiation of RGCs. We identified intrinsic regulator biomarker signatures of these two retinal cell types that can be applied with high confidence to confirm the cell lineage identities.

**Electronic supplementary material:**

The online version of this article (10.1186/s13287-018-0848-7) contains supplementary material, which is available to authorized users.

## Background

The retina is the complex sensory tissue located at the back of an eye and is responsible for transducing light signals into nerve impulses that are conducted by the optic nerve into the visual cortex to form visual perception. The retina is organized into 10 anatomical layers and is composed of several cell types arranged in the anterior–posterior direction, such as retinal ganglion cells (RGCs); bipolar, amacrine and horizontal interneuron cells; Müller glia cells; rod and cone photoreceptor cells; and retinal pigment epithelium (RPE) cells. A number of pathological conditions is associated with the degeneration of different retinal cell types, the most prevalent of them are age-related macular degeneration (AMD), which results from the death of RPE, and glaucoma, associated with the death of RGCs. The RGCs and RPE cells are terminally differentiated cell lineages that cannot be regenerated, and therefore their progressive loss eventually leads to irreversible blindness.

The pluripotent stem cells, such as human embryonic stem cells (hESCs), offer a great potential for application in medicine as they can be directed to differentiate into virtually any cell type of an organism. This makes them an excellent tool for producing relevant cell lineages for tissue repair by transplantation, as well as for in vitro disease modeling and drug testing. The advent of technology for reprogramming somatic cells into induced pluripotent stem cells (iPSCs) by forced overexpression of stemness-associated transcription factors (TFs) further expanded the opportunities for medical application of stem cells [1, 2]. Firstly, the use of human iPSCs (hiPSCs) is not subject to the ethical concerns associated with hESCs, which are obtained from early human embryos. Secondly, using patient-derived hiPSCs allows personalized therapeutic approaches, including the reduction of risks of immune rejection by autologous transplantation of hiPSC-derived cells, as well as feasibility of gene repair for correction of inherited genetic diseases.

Stem cell-based therapy is a promising means for restoration of vision in retinal degenerative disease patients by generating and replacing missing retinal cells [3, 4]. Indeed, various studies have shown that under specifically defined culturing conditions, ESCs and iPSCs can be induced to differentiate along retinal lineages [5]. For example, RPE cells can spontaneously differentiate from ESCs and iPSCs if FGF2 is removed from the culture medium [6, 7]. Subsequently, the efficiency of RPE differentiation was significantly improved by manipulating a number of signaling pathways that contribute to RPE specification during retinogenesis in vivo, which included the use of Wnt and Nodal antagonists [8, 9], TGF-β family member Activin A [10], and a combination of BMP4 antagonist Noggin, FGF2, retinoic acid and sonic hedgehog [11]. In a protocol by Buchholz et al*.* [12], combined application of IGF1, Noggin, Activin A, DKK1, FGF2 and nicotinamide significantly accelerated the time of differentiation and achieved an efficiency of 80%. The clinical trials performed with hESC-derived RPE cells have shown their ability to improve vision in AMD patients and demonstrated a good safety profile [13]. Unlike RPE cells, RGCs require a more complex differentiation procedure, which involves formation of 3D cell aggregates, such as embryoid bodies (EBs) and later optic vesicles (OVs), which are analogous to those formed during eye development in vivo [5]. The modulation of signaling pathways, such as Wnt, IGF1 and TGF-β, using small molecules and growth factors added in a stepwise manner at appropriate times leads to specification of the RGC cell type [5, 14]. In a protocol by Riazifar et al. [15], use of the chemical inhibitor of Notch signaling pathway significantly enhanced efficiency of RGC differentiation from hESCs and hiPSCs.

Differentiation protocols are based on recapitulating the signaling pathways that normally lead to specifications of these cell types in vivo. These extrinsic cues regulate the action of intrinsic regulators, such as TFs and regulators of chromatin state, which in turn execute lineage-specific gene expression programs. At the early stages of eye development, the antagonistic expression of TFs MITF and CHX10 in different regions of OVs specifies the developmental fates toward the RPE and neural retina, respectively [16–18]. Within neural retina, specification of the RGC lineage is initiated by basic helix–loop–helix (bHLH) family TF ATOH7, which in turn regulates expression of TFs BRN3B and ISL1 [19–23]. Different TFs, such as BRN3A, EBF1, EBF3, TBR2, ONECUT1 and ONECUT2, are expressed at the downstream stages of transcriptional cascade [23–26]. By the end, the combinatorial expression of different cell-intrinsic regulators specifies the identity of RGCs and their subtypes [27]. Whereas the process of differentiation of retinal lineages from ESCs or iPSCs mimics development in vivo, differences between these differentiation programs may exist. In order to generate the hiPSC-derived cells that are suitable for medical application, the differentiated cells should conform to the high standards of purity and safety. For this purpose, the expression of signature of markers can be useful.

In this study, we used microarray analysis to characterize the transcriptomes of RPE cells, RGCs and intermediate RGC precursors of OVs, differentiated from the same hiPSC line. We pursued two major objectives: to identify novel potentially important intrinsic regulators of RGC and RPE cell lineage specification in a process of differentiation from hiPSCs; and to identify intrinsic regulator biomarker signatures of these two retinal cell types that can be applied with high confidence to confirm the cell lineage identities. We analyzed two classes of intrinsic regulators: TFs and components of developmentally important epigenetic complexes. We used stringent criteria of the fold-change cutoff values and selected the most drastically differentially regulated genes. Secondly, we applied the criterion of contrasted expression pattern of these between samples to identify the lineage-specific genes as potential candidates for specific biomarkers.

## Methods

### Generation of human induced pluripotent stem cells

Sendai reprogramming vectors (SeV) reprogramming was performed using the CytoTune-iPS 2.0 Sendai Reprogramming Kit (Life Technologies) following the manufacturer’s protocol. Peripheral blood mononuclear cells (PBMCs) were obtained from a healthy male Han Chinese donor and were seeded in 24-well plates (5 × 10^5^ cells/well) in complete PBMC medium. PBMCs were transduced with a mix of SeV vectors encoding OCT3/4, SOX2, KLF4 and c-MYC at a multiplicity of infection (MOI) of 3. The medium was changed every other day, and on day 7 post transduction, 1.25 × 10^5^ cells were plated onto a 10-cm dish previously coated with a mouse embryonic fibroblast (MEF) feeder layer. The day after, the medium was switched to hES medium and the cells were fed every other day for 1 week before switching to the daily feeding. Once the colonies emerged, they were picked by mechanical dissection and transferred to a fresh feeder.

### Differentiation of hiPSCs to RGCs

The protocol of differentiation of hiPSCs to RGCs was adapted with modifications from Riazifar et al. [15]. The confluent hiPSCs were scraped off into small aggregates and transferred to nonadherent dishes containing MTeSR1 medium (STEMCELL Technologies), where they were cultivated for 10 days to generate embryoid bodies (EBs). In the course of cultivation, the medium was replaced every second day with 3:1 (v:v) MTeSR1:human embryonic stem (hES) medium (day 2), 1:1 (v:v) MTeSR1:hES medium (day 4), 1:3 (v:v) MTeSR1:hES medium (day 6) and hES medium (day 8). The hES medium was composed of DMEM/F12 supplemented with 1 mM nonessential amino acids, 1 mM l-glutamate, 1 mM penicillin and 1 mM streptomycin. On the 10th day, EBs were transferred to the plates coated with 0.1% gelatin and were cultivated in hES medium supplemented with 10% FBS and 10 ng/ml IGF for 7 days until neural rosettes (NRs) appeared. Following that, the NRs were scraped off into suspension and cultivated for 12 days in hES medium supplemented with 10% FBS, 2.5 μM retinoic acid (RA) and 10 μM *N*-(*N*-(3,5-difluorophenacetyl)-l-alanyl)-*S*-phenylglycine *t*-butyl ester (DAPT). At this stage the optic vesicles (OVs) have been formed, a sample of which was harvested for RNA isolation and microarray analysis. The OVs were stimulated to differentiate into RGCs by culturing for 1 day in DMEM/F12 containing N-2 supplement (Gibco), on the following day the medium was replaced with Neurobasal medium/B-27 (Gibco) supplemented with DAPT and BDNF, and on the 5th day the medium was replaced with Neurobasal/B-27. On the 15th day the differentiated RGCs were harvested for RNA isolation.

### Differentiation of hiPSCs to RPE cells

The protocol of differentiation of hiPSCs to RPE cells was adapted from Buchholz et al*.* [7]. hiPSCs were passaged onto Geltrex-coated dishes in DMEM/F12 supplemented with N-2 and B-27. From days 0 to 2 this base medium was supplemented with 10 ng/ml IGF1, 50 ng/ml Noggin and 10 ng/ml DKK1. From days 2 to 4 it was supplemented with 10 ng/ml IGF1, 10 ng/ml Noggin, 10 ng/ml DKK1 and 5 ng/ml bFGF. From days 4 to 6 it was supplemented with 10 ng/ml IGF1 and 10 ng/ml DKK1. From days 6 to 8 it was supplemented with 100 ng/m Activin A and 10 μM SU5402. From days 6 to 14 it was supplemented with 100 ng/ml Activin A. On day 14 the foci of the melanin-stained differentiated RPE cells were separated manually from undifferentiated cells and passaged onto a new dish. One more passage was repeated and by day 40 the RPE cells were harvested for RNA extraction and microarray analysis.

### Microarray analysis

Total RNA was purified from the cell lysates of hiPSCs, OVs, RGCs and RPE cells using TRIzol reagent (Invitrogen) according to the manufacturer’s instructions. RNA was analyzed using GeneChip Human Genome U133 Plus 2.0 microarrays (Affymetrix) according to the manufacturer’s instructions. Two biological replicates were analyzed for each cell type. The microarray data analysis was performed in the R (v.3.2.2) environment using Bioconductor packages (http://www.bioconductor.org). The raw intensity data were background corrected, log_2_ transformed and quantile normalized using the Robust Multi-array Average (RMA) algorithm implemented by the affy package [28]. The differentially expressed genes (DEGs) were further identified using the limma package [29]. The probe sets that demonstrated Benjamini–Hochberg adjusted *p* < 0.01 and log_2_ fold-changes of more than 1 or less than −1 (OV vs hiPSC, RGC vs hiPSC and RPE vs hiPSC) were selected for further analysis as DEGs. The microarray data were deposited in the NCBI Gene Expression Omnibus (GEO) database (https://www.ncbi.nlm.nih.gov/geo/) and are accessible via the GEO accession number GSE96853. PANTHER software (version 11.1) was used for statistical overrepresentation analysis (binomial test) of GO-Slim terms, PANTHER Pathways and PANTHER Protein Classes in the lists of DEGs [30].

### Quantitative real-time PCR

cDNA was synthesized from the total RNA samples using SuperScript III Reverse Transcriptase (Invitrogen) and random hexamer primers and was used as a template for SYBR Green qRT-PCR reactions run on the StepOnePlus Real-Time PCR System (Applied Biosystems). The primers used in this study are presented in Additional file 1.

### Immunocytochemistry

For immunofluorescent staining, the cells were washed with PBS and fixed with 4% paraformaldehyde for 30 min at room temperature. After two washes with PBS, the cells were incubated with blocking buffer (10% goat serum and 0.3% Triton X-100 in PBS) for 1 h. Primary antibodies were diluted in blocking buffer and then added to the cells for 2 h at room temperature. The following primary antibodies were used: Bestrophin, MITF, PMEL17, AMPA R2 and Tuj1 (Abcam), ZO-1 (Thermo Fisher Scientific), Math5 (Millipore) and Brn3a (Santa Cruz). The cells were then subjected to three 3-min washes with PBS and incubated with secondary antibodies Alexa Fluor 488 or Alexa Fluor 594 of goat anti-rabbit or goat anti-mouse IgG (Thermo Fisher Scientific) diluted in blocking buffer containing DAPI (Thermo Fisher Scientific) for 1 h at room temperature. After three 3-min washes with PBS, the cells were visualized using confocal microscopy (Zeiss LSM 700).

### Western blot analysis

For western blot analysis, the following primary antibodies were used: PEDF, Islet1 and CECR2 (Santa Cruz), Protein S, Capthesin D, MerTK, AMPA R1, AMPA R2, Brn3a, EBF1 and EBF3 (Abcam), Myosin IIA (Sigma Aldrich), MAP2 (Cell Signaling) and GAPDH (GeneTex). The secondary antibodies used were goat anti-rabbit or goat anti-mouse IgG-HRP (Cell Signaling).

### shRNA-mediated knockdown

The LKO.1-puro-based shRNA constructs targeting *EBF1* were obtained from National RNAi Core Facility (Taipei, Taiwan). The constructs were cotransfected with two viral packaging plasmids, pCMV-d8.91 and pMD.G, into HEK 293 T cells. The virus-containing medium was collected 24, 48 and 72 h after transfection and concentrated by centrifuging at 25,000 rpm for 2 h. The titer of virus was calculated following the method provided by RNAi core (RIU Method) (http://rnai.genmed.sinica.edu.tw/file/protocol/4_1_EstimationLentivirusTiterRIUV1.pdf). The hiPSCs were continuously infected by the virus for 2 weeks followed by puromycin selection until RGC isolation from OVs.

### Electrophysiological analysis

For patch clamp recording, the cell culture medium was replaced with artificial cerebrospinal fluid (125 mM NaCl, 25 mM NaHCO_3_, 1.25 mM NaH_2_PO_4_, 2.5 mM KCl, 25 mM glucose, 2 mM CaCl_2_ and 1 mM MgCl_2_). Cell-attached and whole-cell recordings were performed with patch pipettes (3–5 MΩ) pulled from borosilicate glass tubing (outer diameter 1.5 mm, inner diameter 0.86 mm; Harvard Apparatus, Holliston, MA, USA) filled with internal solution (142 mM K-gluconate, 2 mM KCl, 0.2 mM EGTA, 4 mM MgATP, 10 mM HEPES, 7 mM Na_2_-phosphocreatine, pH 7.3). Signals were recorded with MultiClamp 700B amplifiers or Axopatch 200B amplifiers (Molecular Devices, Union City, CA, USA). Data were filtered at 5 kHz and sampled at 10 kHz with a Digidata 1440A interface (Molecular Devices) controlled by pCLAMP version 10.2 (Molecular Devices). The recording temperature was 22–24 °C.

## Results

### Differentiation of human induced pluripotent stem cells into optic vesicles and retinal ganglion cells

**Fig. 1:**
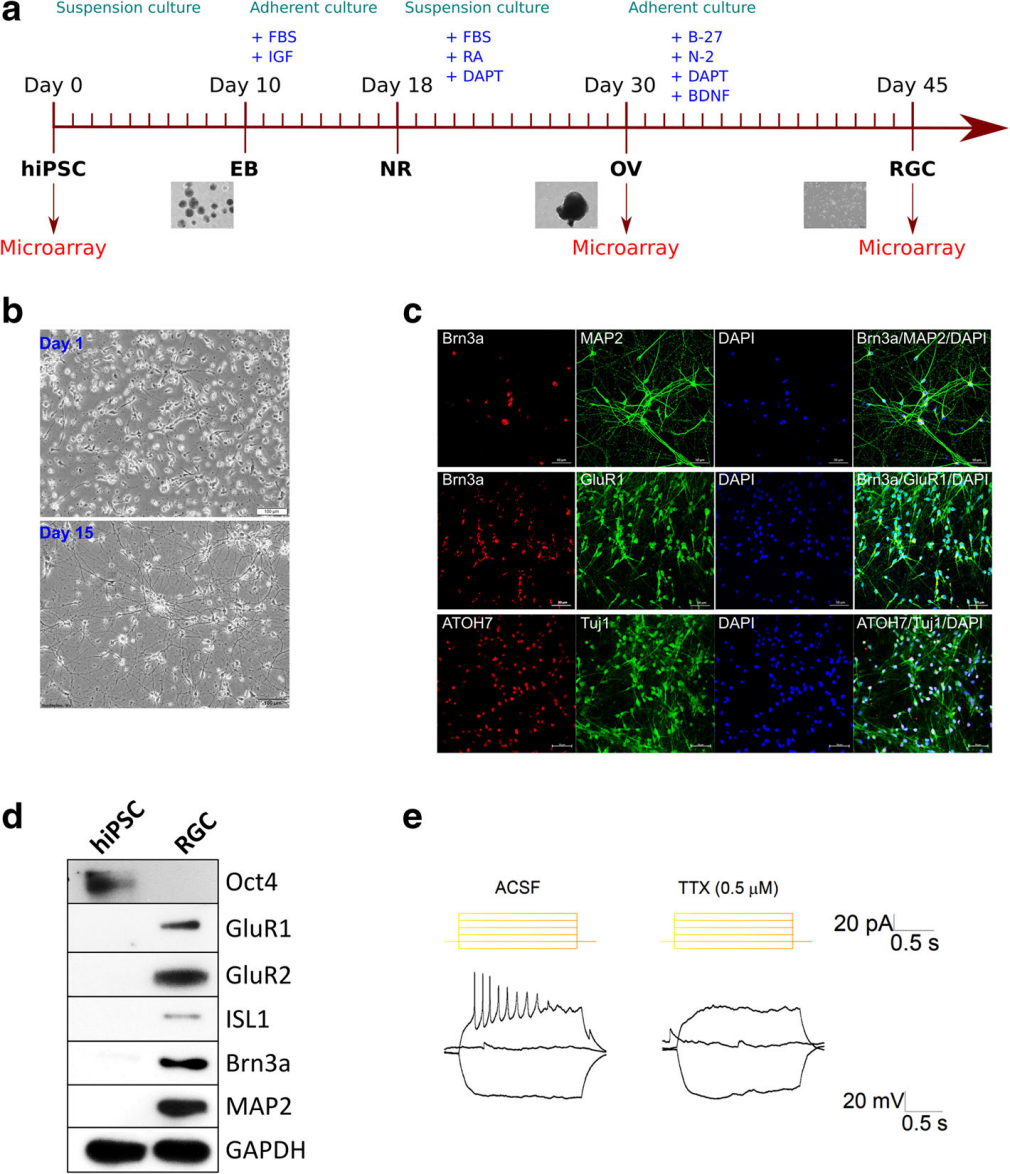
Differentiation of hiPSCs into OVs and RGCs. **a** Schematic of differentiation of hiPSCs into RGCs and time points of sampling for microarray analysis. **b** Bright-field images of RGCs at day 1 of differentiation from OVs, and mature RGCs at 15th day post OVs. **c** Expression of positive RGC markers demonstrated by immunofluorescent microscopy. **d** Western blot analysis demonstrating expression of positive RGC markers in hiPSC**-**derived RGCs. **e** Electrophysiological analysis of differentiated RGCs. Spikes of action potential induced by current application in absence (ACSF) and presence of tetradoxin (TTX). FBS fetal bovine serum, IGF insulin-like growth factor, RA retinoic acid, DAPT *N*-(*N*-(3,5-difluorophenacetyl)-l-alanyl)-*S*-phenylglycine *t*-butyl ester, BDNF brain-derived neurotrophic factor, hiPSC human induced pluripotent stem cell, EB embryoid body, NR neural rosette, OV optic vesicle, RGC retinal ganglion cell, DAPI 4′,6-diamidino-2-phenylindole

In this study, we aimed to analyze transcriptomes of two major terminally differentiated cell lineages of the human retina, RGCs and RPE cells, as well as intermediate retinal progenitor cells (RPCs) of optic vesicles (OVs) derived from hiPSCs. For this purpose, OVs and RGCs were differentiated from hiPSCs in a protocol outlined in Fig. 1a. First, hiPSCs were induced to form embryoid bodies (EBs) by cultivating in a suspension culture. Following that, EBs were grown in the adherent culture containing IGF and FBS until neural rosettes (NRs) were formed. NRs were further induced to form OVs by cultivating with retinoic acid (RA) and Notch signaling pathway inhibitor *N*-(*N*-(3,5-difluorophenacetyl)-l-alanyl)-*S*-phenylglycine *t*-butyl ester (DAPT). RGCs were differentiated from OVs in the course of 15 days, during which they developed their characteristic morphology of long neurite outgrowth (Fig. 1b). The identity of the differentiated RGCs was further verified by detecting expression of the known RGC markers BRN3A, MAP2, GluR1, ATOH7 and Tuj1 by immunofluorescent staining (Fig. 1c) and markers GluR1, GluR2, ISL1, BRN3A and MAP2 by western blot analysis (Fig. 1d). At the same time, expression of stemness marker Oct4 was completely ablated in hiPSC-derived RGCs in contrast to original hiPSCs (Fig. 1d). The electrophysiological properties of the differentiated RGCs were tested by the patch clamp technique, whereby it was shown that current injection caused firing of action potential, thus indicating the functional maturity of the neurons (Fig. 1e).

### Differentiation of hiPSCs into RPE cells

**Fig. 2:**
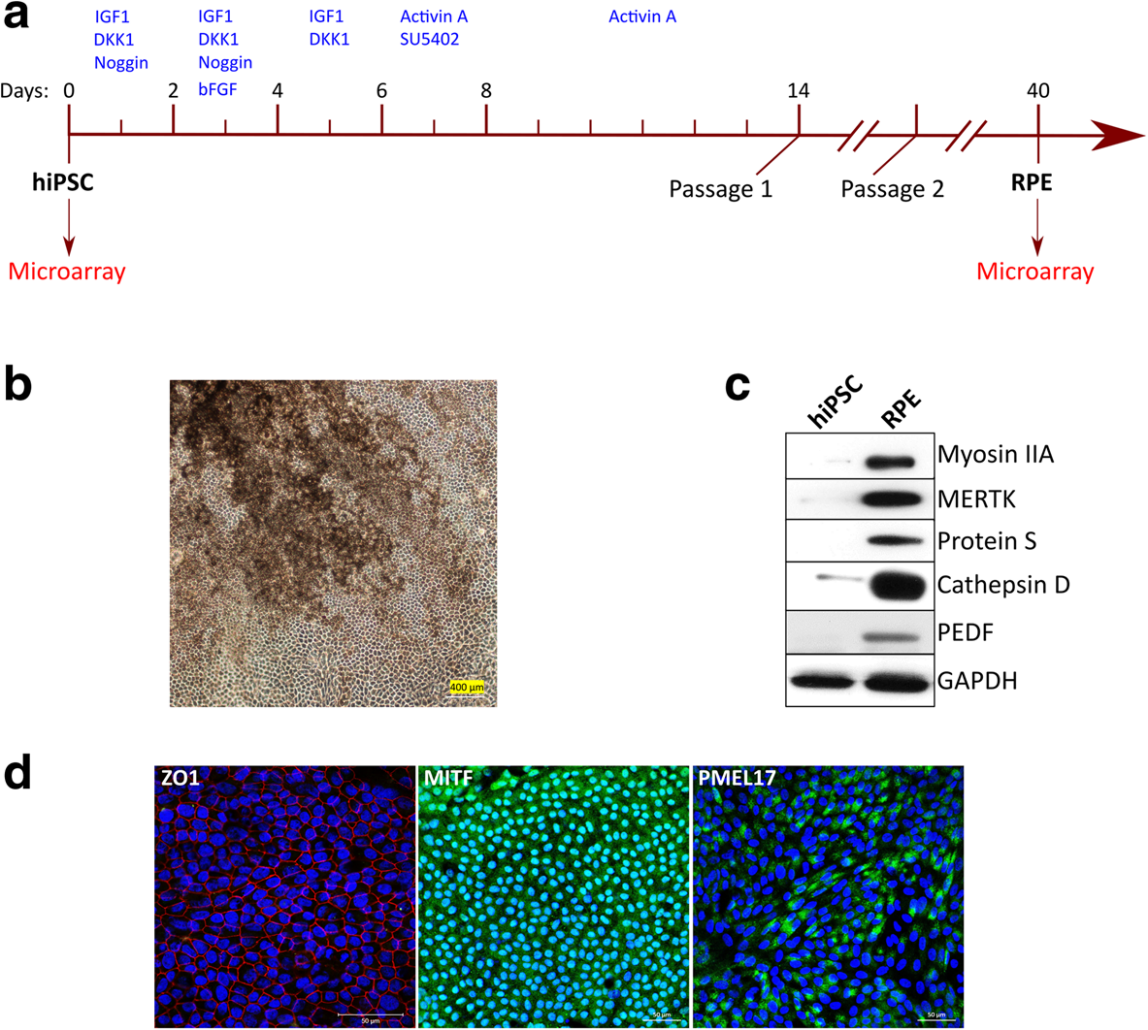
Differentiation of hiPSCs into RPE cells. **a** Schematic of differentiation of hiPSCs into RPE cells and time points of sampling for microarray analysis. **b** Bright-field image of hiPSC-derived RPE cells, showing melanin pigmentation. **c** Western blot analysis demonstrating expression of positive RPE markers in hiPSC**-**derived RPE cells. **d** Expression of positive RPE markers demonstrated by immunofluorescent microscopy. Nuclei counterstained with DAPI, and merged DAPI and immunofluorescent signals shown. IGF insulin-like growth factor, bFGF basic fibroblast growth factor, hiPSC human induced pluripotent stem cell, RPE retinal pigment epithelium

RPE cells were derived from hiPSCs in a process of differentiation directed by a number of morphogenic factors, such as IGF1, DKK1, Noggin, Activin A and SU5402, added at appropriate times (Fig. 2a). As expected, the differentiated RPE cells were stained with melanin pigment when examined by bright-field microscopy (Fig. 2b). Expression of the characteristic RPE markers myosin IIA, MERTK, protein S, cathepsin D and PEDF was confirmed by western blot analysis (Fig. 2c) and expression of PMEL17, MITF and ZO-1 was confirmed by immunofluorescent staining (Fig. 2b).

### Identification of genes differentially expressed in OV, RGC and RPE lineages

**Fig. 3:**
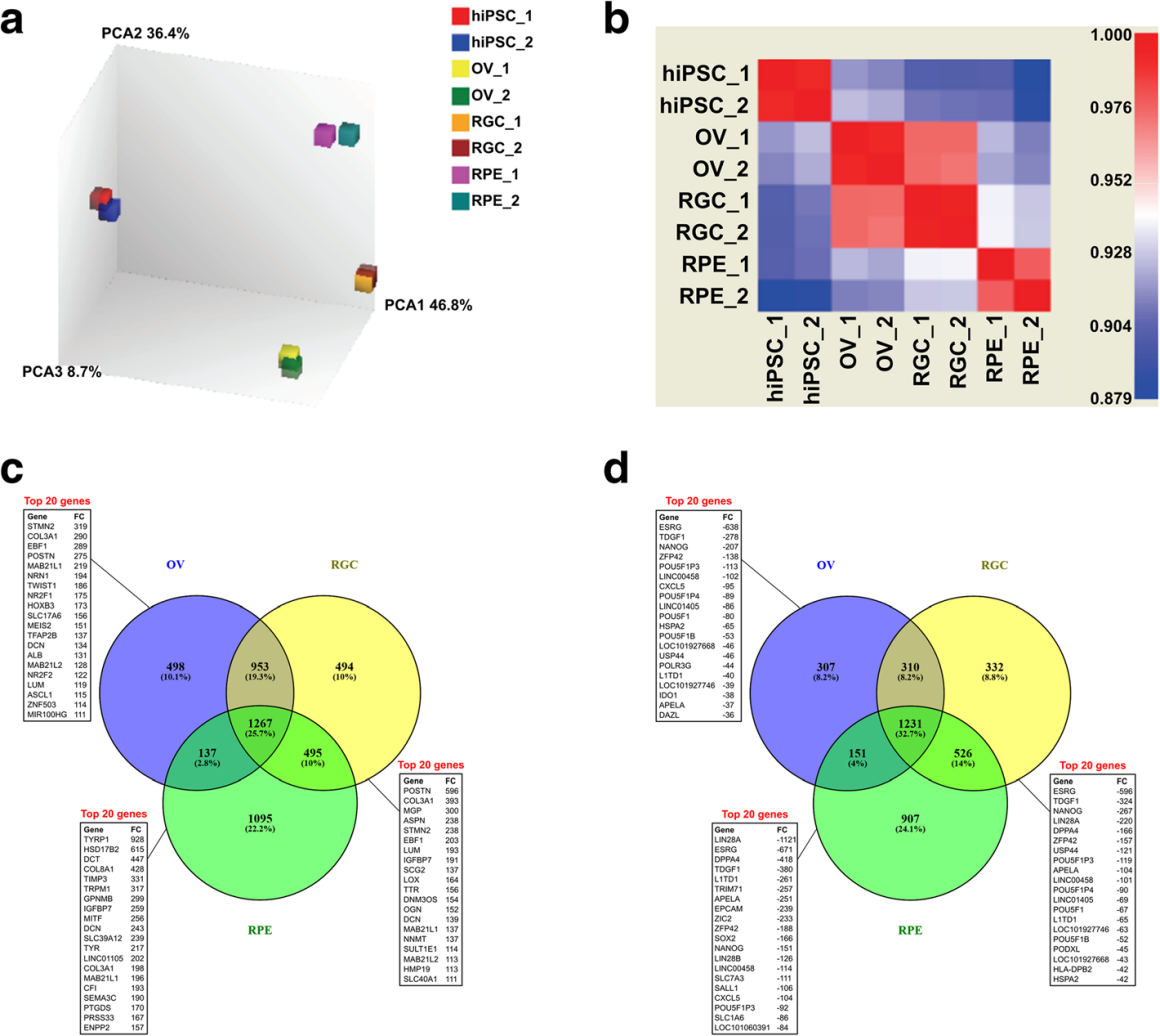
Microarray analysis of transcriptomes of OV, RGC and RPE samples derived from hiPSCs. **a** Principal component analysis (PCA) of hiPSC, OV, RGC and RPE biological replicate samples. **b** Pearson correlation matrix of hiPSC, OV, RGC and RPE biological replicate samples. **c** Venn diagram demonstrating number of common and unique differentially overexpressed genes in OV, RGC and RPE samples compared to original hiPSCs according to criteria of linear fold-change > 2 and *p* < 0.01. Text boxes show top 20 DEGs sorted by fold-change in each sample. **d** Venn diagram demonstrating number of common and unique differentially underexpressed genes in OV, RGC and RPE samples compared to original hiPSCs according to criteria of linear fold-change > 2 and p < 0.01. Text boxes show top 20 DEGs sorted by fold-change in each sample. hiPSC human induced pluripotent stem cell, OV optic vesicle, RGC retinal ganglion cell, RPE retinal pigment epithelium

Total RNA isolated from hiPSCs, OVs, RGCs and RPE cells was analyzed using Affymetrix U133 Plus 2.0 microarrays. Principal component analysis (PCA) and Pearson correlation analysis demonstrated that there was low variability between biological replicate samples (Fig. 3a, b). The genes differentially expressed in OV, RGC and RPE lineages relative to the original hiPSCs were selected according to the criteria of FDR-adjusted *p* < 0.01 and the log_2_ expression fold-change being more than 1 or less than −1 for positively and negatively expressed genes, respectively. According to these criteria, a total of 2855 genes were upregulated in OVs, 3209 genes in RGCs and 2994 genes in RPE cells as compared with the original hiPSCs (Fig. 3c). Among the upregulated genes, 1267 were common between all three lineages (Fig. 3c). Similarly, 1999 genes were downregulated in OVs, 2399 genes in RGCs and 2815 genes in RPE cells, with 1231 of downregulated genes being common among all three lineages (Fig. 3d). Among all of the differentially expressed genes for all three lineages, the RPE lineage had the highest percentage of unique DEGs (22.2% and 24.1% for positively and negatively expressed genes, respectively), emphasizing its furthest divergence from the original hiPSC line (Fig. 3c, d). Among the top 20 overexpressed genes were those encoding developmentally important TFs, such as *EBF1*, *TWIST1*, *NR2F1*, *HOXB3* and *MEIS2* in OVs, *EBF1* in RGCs and *MITF* in RPE cells (Fig. 3c). Among the top 20 underexpressed genes were mostly factors involved in maintenance of embryonic stem cells, such as *NANOG*, *POU5F1* (*OCT4*), *SOX2* and *LIN28A* (Fig. 3d). As could be expected, among the top 20 overexpressed genes in the RPE lineage were *TYRP1*, *DCT* and *TYR*, encoding enzymes involved in biosynthesis of melanin: tyrosinase related protein 1, dopachrome tautomerase and tyrosinase, respectively.

### Functional enrichment analysis

**Fig. 4:**
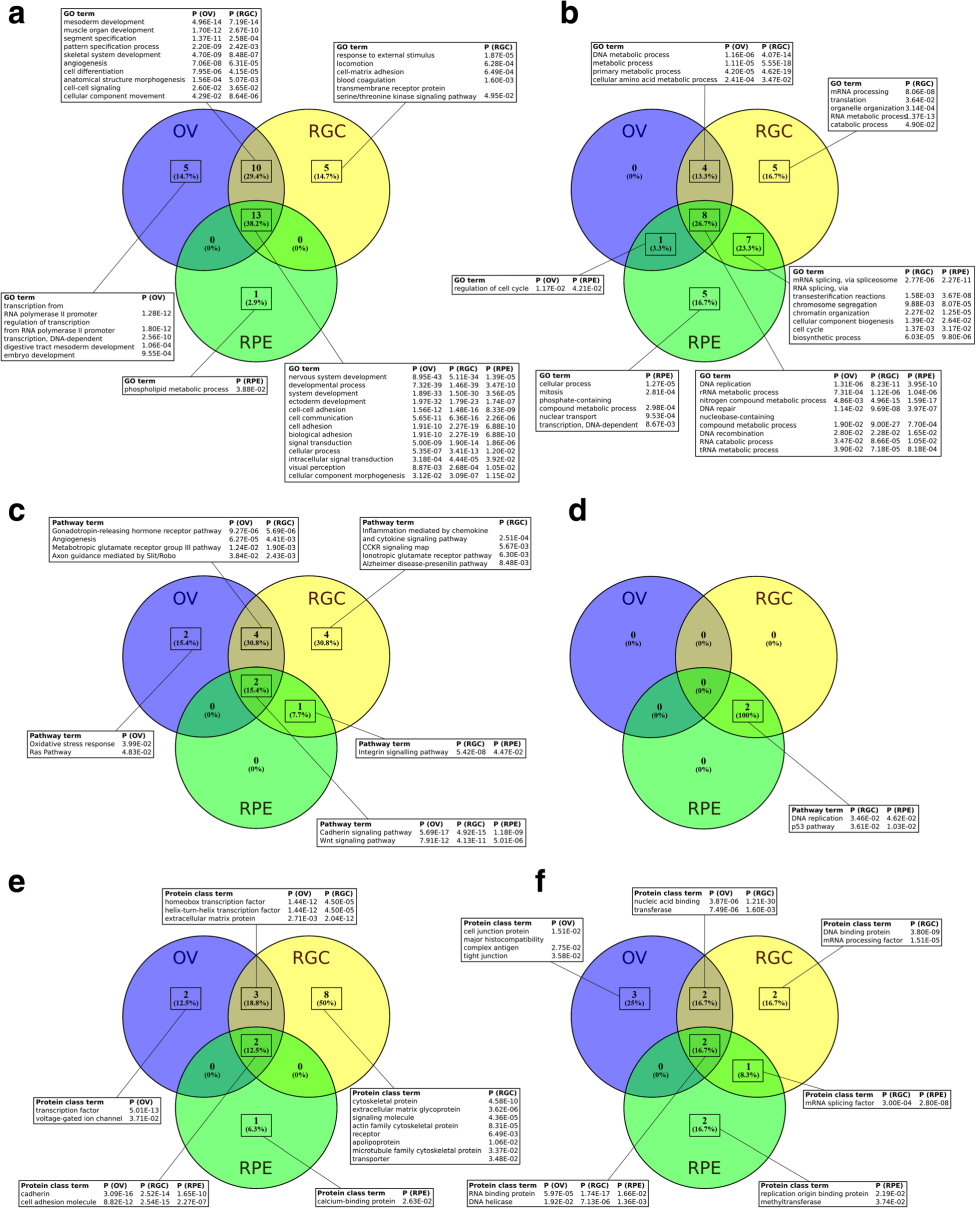
Functional enrichment analysis of upregulated and downregulated DEGs according to PANTHER classification system. Data presented as Venn diagrams, showing common and unique enriched terms among OV, RGC and RPE lineages. Terms with respective *p* values listed in text boxes. **a** Gene Ontology Slim (GO-Slim) Biological Process terms enriched among upregulated DEGs. **b** GO-Slim Biological Process terms enriched among downregulated DEGs. **c** PANTHER Pathway terms enriched among upregulated DEGs. **d** PANTHER Pathway terms enriched among downregulated DEGs. **e** PANTHER Protein Class terms enriched among upregulated DEGs. **e** PANTHER Protein Class terms enriched among downregulated DEGs. OV optic vesicle, RGC retinal ganglion cell, RPE retinal pigment epithelium

The lists of DEGs were analyzed for enrichment of Gene Ontology Slim (GO-Slim) Biological Process (BP), PANTHER Pathway and PANTHER Protein Class terms according to the PANTHER classification system (Fig. 4). As expected, the most enriched BP terms among the upregulated genes common between OVs, RGCs and RPE cells were related to development: the top three terms with the lowest *p* values in the OV list were “nervous system development”, “developmental process” and “system development” (Fig. 4a). Similarly, among the genes common between OVs and RGCs, but not RPE cells, the top terms were “mesoderm development”, “muscle organ development” and “segment specification” (Fig. 4a). The second group of the most enriched terms in the list of upregulated DEGs common between OVs, RGCs and RPE cells was related to cell adhesion: “cell–cell adhesion”, “cell communication” and “cell adhesion” (Fig. 4a). The top enriched BP terms unique to OVs were related to transcription, the top three being “transcription from RNA polymerase II promoter”, “regulation of transcription from RNA polymerase II promoter” and “transcription, DNA-dependent”. In the list of upregulated genes unique for RGCs, the top three BP terms were “response to external stimulus”, “locomotion” and “cell matrix adhesion” (Fig. 4a). The only enriched BP term unique for RPE cells was “phospholipid metabolic process” (Fig. 4a). Among the downregulated genes, the most enriched BP GO-Slim terms were mostly related to the core mechanisms of cell proliferation, such as “DNA replication”, “rRNA metabolic process” and “mRNA splicing, via spliceosome” (Fig. 4b). The most enriched PANTHER pathways common between upregulated genes in OVs, RGCs and RPE cells were “cadherin signaling pathway” and “Wnt signaling pathway”, and common between RGCs and RPE cells was “integrin signaling pathway” (Fig. 4c). The only PANTHER pathways enriched in the downregulated gene list were “DNA replication” and “p53 pathway”, both common between RGCs and RPE cells (Fig. 4d). The most enriched PANTHER Protein Class terms in the upregulated gene list common between OVs, RGCs and RPE cells were “cadherin” and “cell adhesion molecule”, and in the list common between OVs and RGCs were “homeobox transcription factor”, “helix-turn-helix transcription factor” and “extracellular matrix protein” (Fig. 4e). The top Protein Class term unique for OVs was “transcription factor”, unique for RGCs was “cytoskeletal protein” and unique for RPE cells was “calcium-binding protein” (Fig. 4e). The downregulated genes were mostly enriched in Protein Class terms typical for actively proliferating cells, such as “RNA binding protein”, “nucleic acid binding”, “DNA binding protein” and “replication origin binding protein” (Fig. 4f).

### Differentially regulated transcription factors

**Fig. 5:**
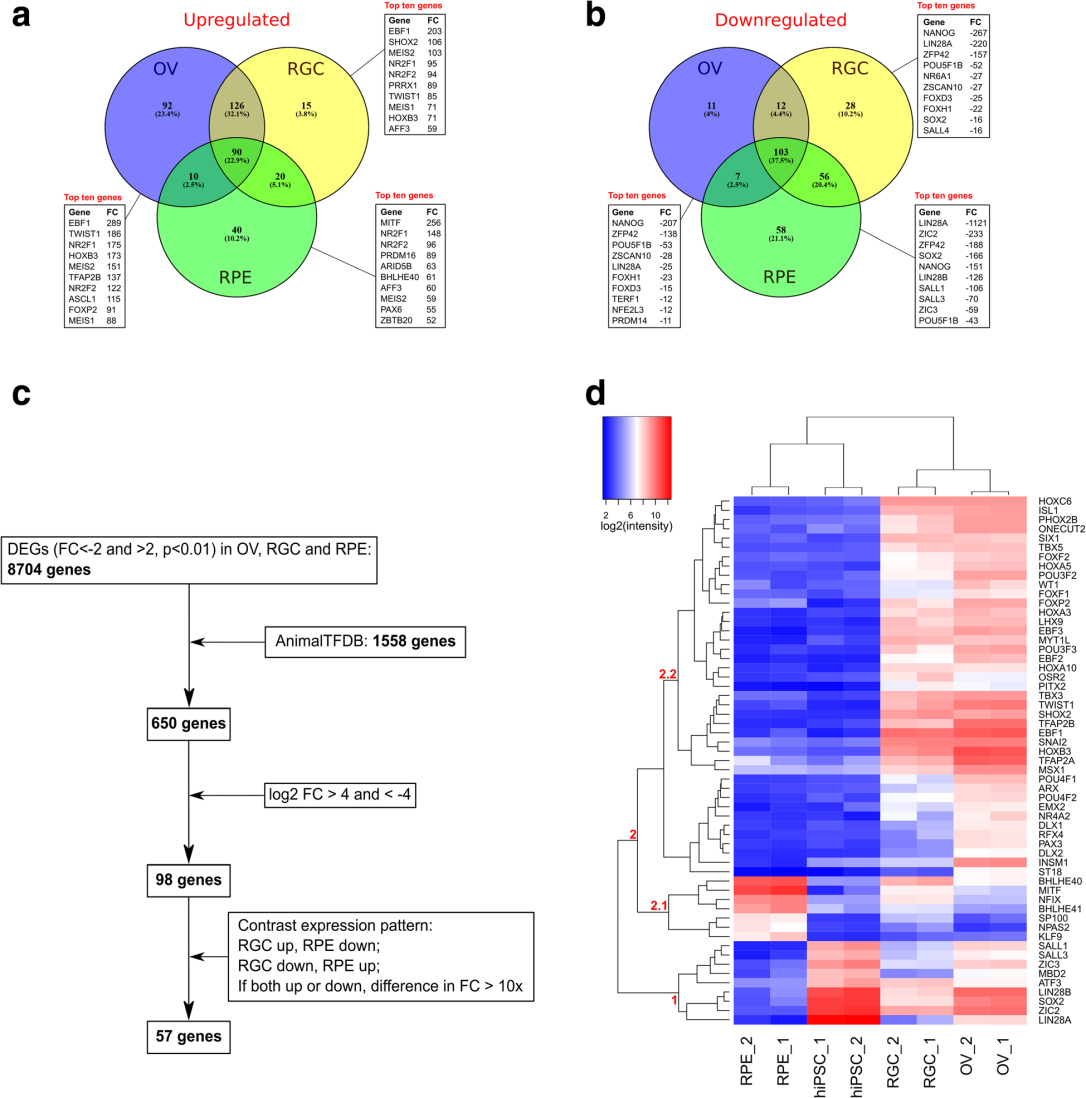
Differentially expressed transcription factors (TFs). Venn diagrams demonstrate numbers of common and unique differentially overexpressed (**a**) and underexpressed (**b**) TF-encoding genes in OV, RGC and RPE lineages compared to original hiPSCs according to criteria of fold-change > 2 and *p* < 0.01. Top 10 most highly differentially expressed TF-encoding genes with respective fold-changes shown in text boxes. **c** Flowchart demonstrating selection of 57 most highly regulated TF-encoding genes with contrast expression patterns between samples. **d** Hierarchical clustering of the selected 57 TF-encoding genes. Three clusters (1, 2.1 and 2.2) described in Results are labeled red. OV optic vesicle, RGC retinal ganglion cell, RPE retinal pigment epithelium, FC fold-change, DEG differentially expressed gene, AnimalTFDB Animal Transcription Factor Database

The main objective of our study was to characterize the retinal lineage differentiation from hiPSCs in terms of differential expression of regulatory genes in order to elicit new potential key intrinsic regulators. Given the fact that transcription factors (TFs) represent the major class of intrinsic developmental regulators, we described the differential expression of 1558 TFs catalogued in the Animal Transcription Factor Database (http://bioinfo.life.hust.edu.cn/AnimalTFDB/). Among the TF-encoding genes differentially expressed according to the log_2_ fold-change cutoff value of more than 1 or less than −1 with FDR-adjusted *p* < 0.01, there were a total of 318 genes overexpressed in OVs, 251 genes in RGCs and 160 genes in RPE cells, with 90 genes being common among all three lineages (Fig. 5a). At the same time, 133 genes were underexpressed in OVs, 199 genes in RGCs and 224 genes in RPE cells, with 103 downregulated genes being common among all three lineages (Fig. 5b). In order to narrow down the list of TFs that may play a particularly important regulatory role in discriminating between the developmental paths leading toward RGC versus RPE lineages, we applied two major criteria. Firstly, we used a very stringent fold-change cutoff value (log_2_ FC > 4 and < −4) to select the 98 most drastically regulated genes out of a total of 650 differentially expressed TF-encoding genes (Fig. 5c). Secondly, out of these 98 genes, we selected 57 according to the criterion of contrasted expression pattern between OV, RGC and RPE samples, which we defined as upregulated in one sample and downregulated in another sample; and in the case of the genes upregulated or downregulated at the same time in all samples, we selected those in which the difference in fold-changes between any two samples was more than 10-fold (Fig. 5c). These 57 genes were organized into three major clusters according to hierarchical clustering, as shown in Fig. 5d: cluster 1 included nine genes downregulated in all samples, to a larger extent in RPE cells and to a lesser extent in OVs; cluster 2.1 included seven genes most drastically upregulated in RPE cells and marginally upregulated in OVs and RGCs; and cluster 2.2 included the majority of genes, which were most highly upregulated in OVs/RGCs, but at the same time downregulated, not regulated or only marginally upregulated in RPE cells. Importantly, among the seven RPE-overexpressed genes of cluster 2.1 was *MITF1*, the well-known master regulator of RPE lineage specification; and among the OV/RGC-overexpressed genes of cluster 2.2 were *ISL1*, *POU4F1* and *POU4F2*, the well-characterized master regulators of RGC development (Fig. 5d).

### Validation of differentially expressed TF-encoding genes by qRT-PCR

**Fig. 6:**
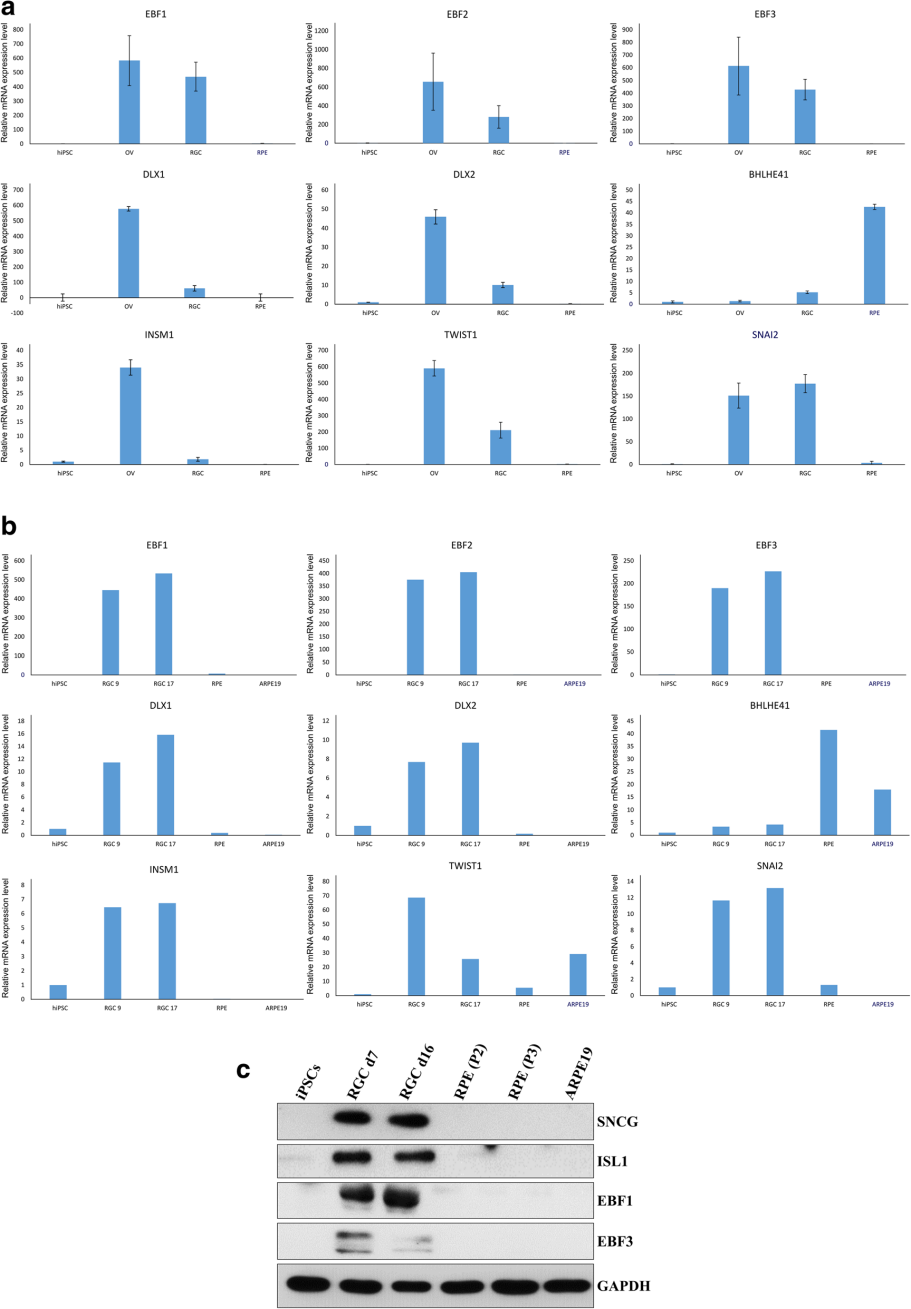
Validation of microarray results by qRT-PCR and western blot analysis. **a** qRT-PCR testing expression of selected TF-encoding mRNAs using the same total RNA as analyzed by microarray. Data for OVs, RGCs and RPE cells presented as fold-changes relative to mRNA level in hiPSC, standard deviation between replicates shown as error bars. **b** qRT-PCR testing expression of selected TF-encoding mRNAs using RNA obtained from independent set of cells: hiPSCs, RGCs after 9 days of differentiation from OVs (RGC 9), RGCs after 17 days of differentiation from OVs (RGC 17), hiPSC-derived RPE cells (RPE) and RPE cell line ARPE-19 (ARPE19). Data presented as fold-changes relative to hiPSC. **c** Western blot analysis demonstrating overexpression of EBF1 and EBF3 TFs in RGCs, but not in RPE cells and hiPSCs, similarly to well-known RGC markers SNCG and ISL1. iPSC induced pluripotent stem cell, RGC retinal ganglion cell, RPE retinal pigment epithelium

Some of the TF-encoding genes with the pronounced differential expression in RGCs and RPE cells were selected for validation of microarray results by qRT-PCR. For qRT-PCR validation, we used the same total RNA that was used for microarray analysis (Fig. 6a). In addition, in order to test biological reproducibility of the expression patterns revealed by microarray, we used RNA extracted from the independent set of cells that included RGCs at different stages of maturity (9 days and 17 days post OV stage), hiPSC-derived RPE cells and a commercial ARPE-19 cell line (Fig. 6b). Remarkably, the most highly overexpressed gene in OVs and RGCs, but not in RPE cells, was *EBF1*, which encodes the member of Collier/Olfactory-1/Early B-cell (COE) family of TFs (Fig. 6a, b). Two of its paralogs, *EBF2* and *EBF3*, also demonstrated the same pattern of expression (Fig. 6a, b). *DLX1*, *DLX2* and *INSM1* were among the genes whose expression level was maximal in OVs, elevated to a lesser extent in differentiated RGCs and significantly downregulated in RPE, which indicates their important role as regulators of commitment of OV RPCs toward the RGC lineage (Fig. 6a, b). TF-encoding gene *BHLHE41* was confirmed to be considerably upregulated in RPE and ARPE-19, but only marginally in RGCs (Fig. 6a, b), indicating its potential role in RPE development. *TWIST1* and *SNAI2*, encoding TFs important for epithelial–mesenchyme transition, were more upregulated in OVs and RGCs than in RPE cells (Fig. 6a, b), although the former was quite highly expressed in ARPE-19 (Fig. 6b). Elevated expression of EBF1 and EBF3 proteins in hiPSC-derived RGCs was confirmed by western blot analysis (Fig. 6c).

### Knockdown of *EBF1* leads to defective RGC differentiation

**Fig. 7:**
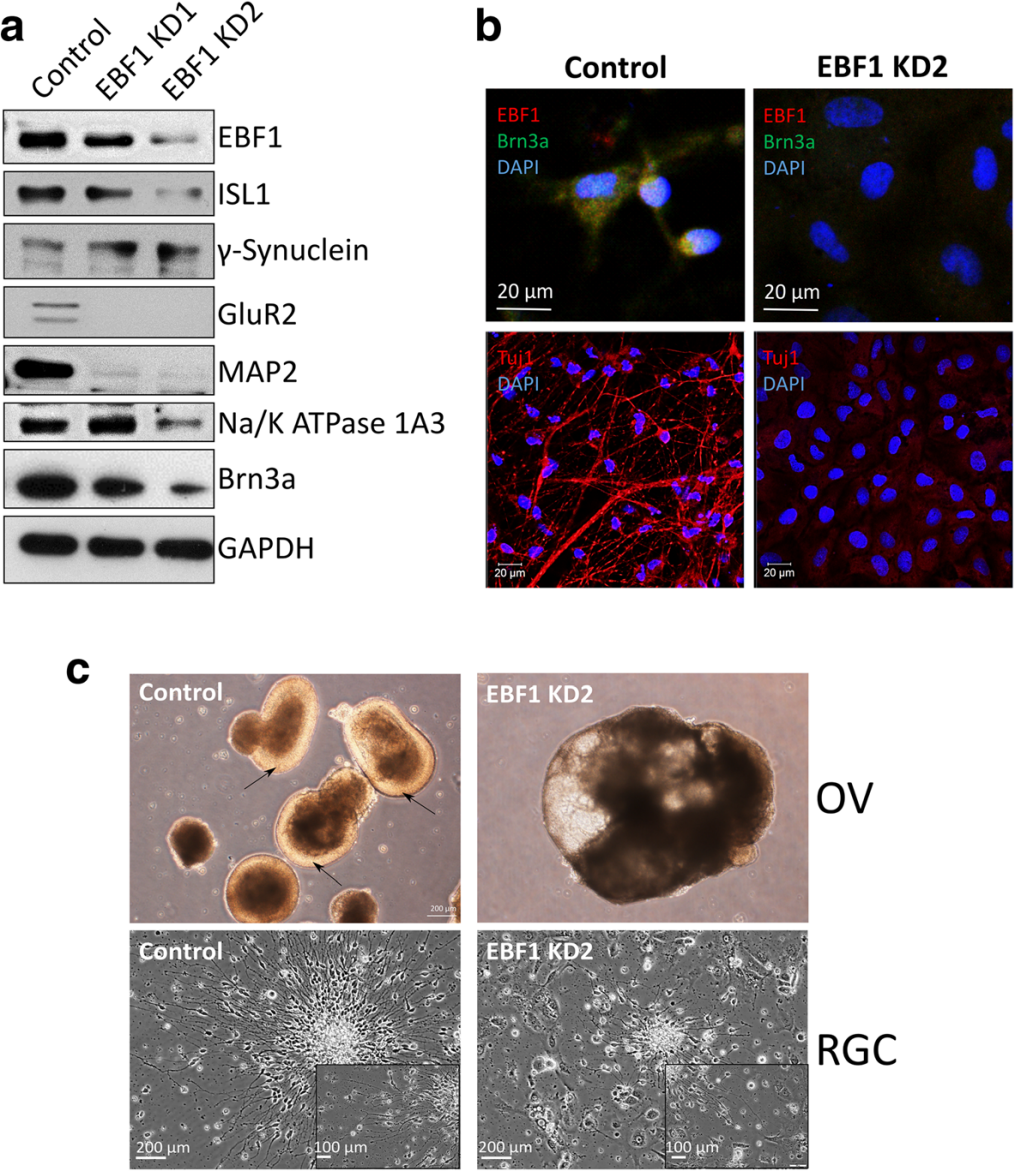
Effect of knockdown of *EBF1* on differentiation of RGCs. **a** Western blot analysis demonstrating expression levels of EBF1 and indicated RGC markers in RGCs differentiated from control and *EBF1* knockdown (EBF KD1 and EBF KD2) hiPSCs. GAPDH used as loading control. **b** Immunofluorescent microscopy showing expression of EBF1, BRN3A and Tuj1 in control and *EBF1* knockdown RGCs (EBF1 KD2). **c** Bright-field microscopy demonstrating morphology of OVs (top) and RGCs (bottom) derived from control and *EBF1* knockdown (EBF1 KD2) hiPSCs. Arrows indicate neural retina precursor layer of brighter color. DAPI 4′,6-diamidino-2-phenylindole, OV optic vesicle, RGC retinal ganglion cell

Since *EBF1* was the most highly overexpressed TF-encoding gene in the OV and RGC lineages, we tested its role in differentiation by performing functional knockdown. We designed two shRNA lentiviral constructs targeting *EBF1* (EBF1 KD1 and EBF1 KD2), infected hiPSCs, selected for stable knockdown of hiPSC clones, and induced their differentiation into OVs/RGCs according to the protocol outlined in Fig. 1a. Western blot analysis at day 32 post induction of differentiation revealed depletion of EBF1 protein by both shRNA constructs, with higher efficiency by EBF1 KD2 (Fig. 7a). At the same time, the levels of ISL1 and BRN3A, the TFs crucial for RGC lineage specification, were noticeably decreased, as were the RGC markers GluR2, MAP2 and Na/K ATPase 1A3 (Fig. 7a). This depletion was more pronounced in more efficient EBF1 KD2 knockdown (Fig. 7a). On the other hand, the level of the RGC marker γ-synuclein was not decreased by *EBF1* knockdown (Fig. 7a). Similarly, immunofluorescence staining of EBF1 KD2 cells demonstrated efficient knockdown of EBF1 and concomitant depletion of RGC markers BRN3A and Tuj1 (Fig. 7b). EBF1 KD2 cells demonstrated abnormal differentiation and morphology at the OV stage: firstly, the EBF1 KD2 OVs were of bigger size than normal control OVs; secondly, they did not form the neural retina precursor layer (Fig. 7c). Whereas the control hiPSCs by day 32 of differentiation formed a homogeneous population of RGCs with neuronal morphology, differentiation of EBF1 KD2 cells resulted in a mixed population of cells, with very few of them having neuronal morphology (Fig. 7c).

### Differentially regulated epigenetic factors

**Fig. 8:**
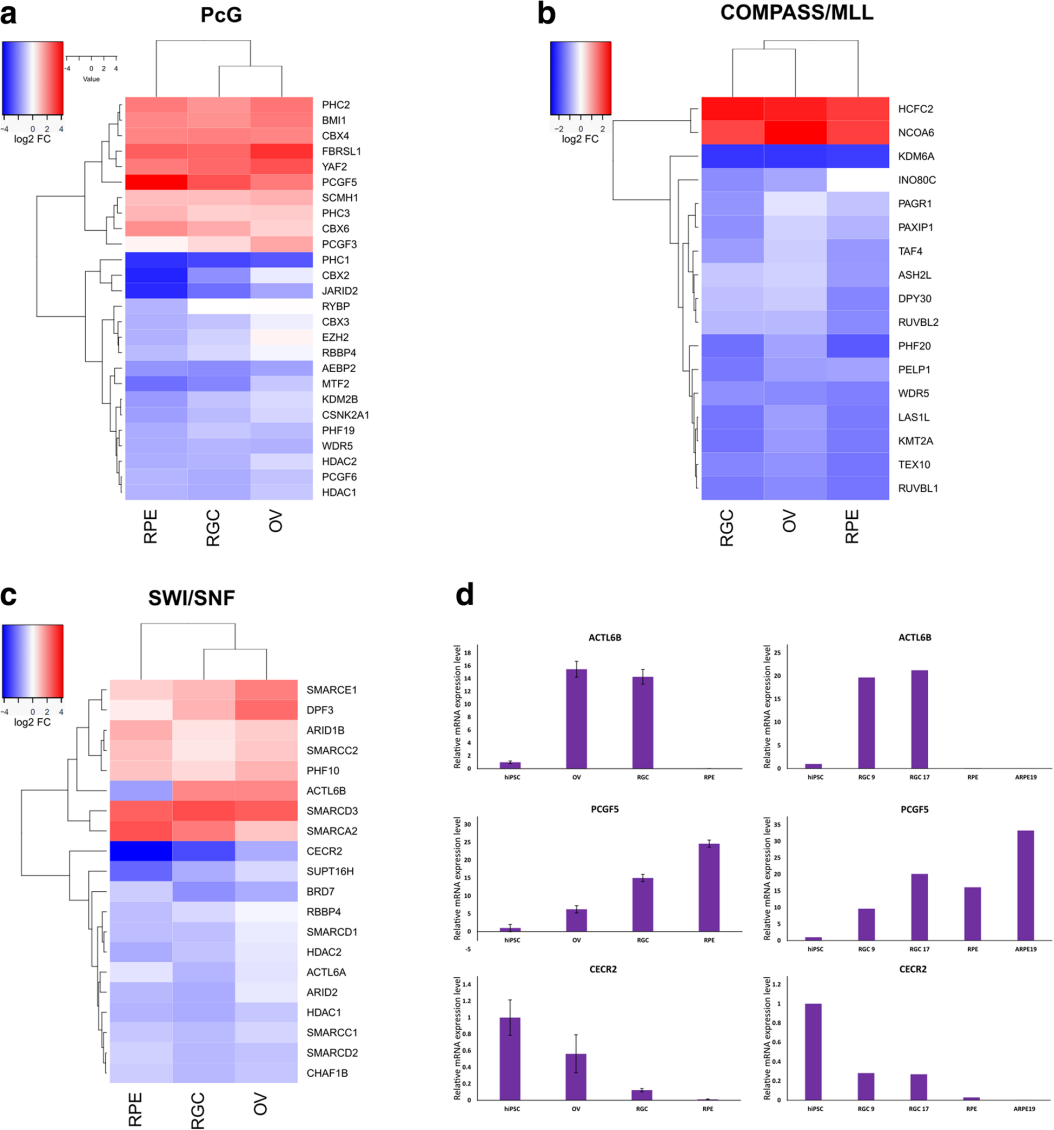
Differentially expressed components of epigenetic complexes. **a** Hierarchical clustering of genes encoding components of PcG complexes. **b** Hierarchical clustering of genes encoding components of COMPASS/MLL complexes. **c** Hierarchical clustering of genes encoding components of SWI/SNF complexes. **d** qRT-PCR validation of expression patterns of *ACTL6B*, *PCGF5* and *CECR2* genes using same RNA as used for microarray (left) and RNA obtained from independent set of cells (right) that included two stages of differentiation of RGCs (9 and 17 days post OV), hiPSC-derived RPE cells (RPE) and RPE cell line ARPE-19. FC fold-change, OV optic vesicle, RGC retinal ganglion cell, RPE retinal pigment epithelium

Epigenetic regulation of the chromatin state by various multisubunit chromatin modifying and chromatin remodeling complexes constitutes an important mechanism for cell lineage commitment and differentiation. Two groups of such complexes, Trithorax group (TrxG) and Polycomb group (PcG), have antagonistic effects on transcriptional activity associated with the chromatin state: the former causes activation and the latter causes silencing of transcription [31]. Therefore, we analyzed differential expression of various components of the key PcG complexes, Polycomb repressive complexes 1 (PRC1) and 2 (PRC2), as well as two main families of TrxG complexes: COMPASS/MLL and SWI/SNF (BAF/PBAF). For this purpose, we used the EpiFactors Database [32] to retrieve the gene names, whose products are annotated as components of the respective complexes, and analyzed their differential expression in the microarray. As shown in Fig. 8a, some components of the PcG complexes were upregulated, and a slight majority was downregulated. Notably, the *PCGF5* gene was more highly overexpressed in RPE cells than in OVs and RGCs, and the *CBX2* gene was more considerably underexpressed in RPE cells, both genes being components of the PRC1 complex (Fig. 8a). The vast majority of the components of COMPASS/MLL complexes were underexpressed in all samples and only two (*HCFC2*, *NCOA6*) were overexpressed (Fig. 8b). Similarly, 40% of genes encoding components of the SWI/SNF complex were overexpressed, and the rest were underexpressed (Fig. 8c). Some of the genes encoding the components of epigenetic complexes that demonstrated noticeably contrasted expression patterns between different samples were selected for qRT-PCR validation (Fig. 8d). For this purpose we used the same RNA that was used for microarray analysis, as well as RNA extracted from the independent set of cells that included RGCs at different stages of maturity (9 days and 17 days post OV stage), hiPSC-derived RPE cells and the commercial ARPE-19 cell line (Fig. 8d). *ACTL6B*, encoding the component of the SWI-SNF nBAF complex, was upregulated in RGCs, but at the same time was significantly underexpressed in both RPE and ARPE-19 cells (Fig. 8c, d). *CECR2*, another gene encoding the SWI/SNF component, demonstrated an interesting pattern of expression, as it was marginally underexpressed in OVs, more significantly in RGCs and very significantly in RPE cells (Fig. 8c, d). The PRC1 component-encoding gene *PCGF5* demonstrated reversed pattern of expression, with relatively slight overexpression in OVs and early RGCs (9 days), and more drastic overexpression in mature RGCs and RPE cells (Fig. 8c, d).

## Discussion

In recent years, several protocols have been developed to differentiate retina cell lineages from pluripotent stem cells, such as ESCs and iPSCs, which are based on recapitulating the pathways that lead to specification of retinal cell types during the natural development of an eye in an embryo [5]. The bifurcation of the neural retina lineage from primordial retinal pigment epithelium is the key event that occurs in the early stages during eye development that gives rise to retinal neurons and RPE [33]. These extrinsic cues regulate the action of intrinsic regulators, such as transcription factors (TFs) and regulators of chromatin state, which in turn execute lineage-specific gene expression programs. Some of these intrinsic regulators of normal eye development are well known, such as BRN3A, BRN3B and ATOH7 during RGC development and MITF during RPE development. These factors are widely used as markers to confirm specificity of cell lineages derived from iPSCs. However, their expression is not limited to the given cell type only, and therefore it is important to analyze markers in combination to define a particular cell type with high specificity. In addition, development of lineages from iPSCs can be different and involve different intrinsic regulator networks as compared to normal eye development, which may result in similar, but not equivalent, cell types. Therefore, in this study we focused on characterizing the expression patterns of various intrinsic regulators in the process of differentiation of hiPSCs into RGCs and RPE cells in order to expand the repertoire of markers for these hiPSC-derived cell types and reveal new potential key regulators of their differentiation.

In this study, we followed the most recent and efficient protocols to differentiate hiPSCs into RGCs and RPE cells [7, 15]. The identity of these cell lines was confirmed by demonstrating expression of their well-known markers by western blotting analysis and immunostaining (Figs. 1 and 2). Gene expression profiling revealed that differentiation was associated with drastic differential gene expression (Fig. 3). Functional enrichment analysis demonstrated several general patterns, such as overrepresentation of genes involved in DNA replication and cell cycle in the lists of underexpressed DEGs, which is consistent with these processes being the signatures of actively proliferating pluripotent stem cells (Fig. 4b, d, f). On the other hand, overexpressed genes common to all lineages were mostly enriched in TF-encoding genes and genes encoding cell adhesion proteins, particularly the cadherins (Fig. 4a, c, e), which is consistent with the crucial roles of TFs as intrinsic regulators of differentiation, and cadherins as key determinants of tissue morphogenesis [34]. Remarkably, TF-encoding genes were mostly overrepresented in the upregulated transcriptome of OVs, which may reflect the heterogeneity of cell types and their different transcriptional programs, constituting this intermediate organoid. To narrow down the lists of potential key regulators of retinogenesis, we focused our microarray analysis on expression patterns of the intrinsic regulators, which we classified into two classes: TFs and epigenetic regulators. We applied two criteria for the selection of potentially important regulators and markers: fold-change of upregulation, and the contrasted pattern of differential expression between OV, RGC and RPE lineages.

For analysis of TFs, we used annotation by the AnimalTF database [35]. Interestingly, the topmost overexpressed TFs in OV retinal progenitor cells and RGCs were three closely related paralogs of the Collier/Olfactory-1/Early B-cell (COE) family of TFs (*EBF1*, *EBF2* and *EBF3*), which demonstrated on average 300–800-fold upregulation in OV and RGC lineages, as compared to the original hiPSCs, but at the same time were mostly downregulated in RPE cells. This is consistent with the study by Trimarchi et al. [36], who demonstrated by single cell microarray profiling that *Ebf3* is one of the most highly specific biomarkers of the mouse RGCs. Later, it was shown that all four members of the COE family are expressed in mouse RGCs, and transcription of the *Ebf1, Ebf2* and *Ebf3* genes is regulated by BRN3B and is required but not sufficient for RGC differentiation [37]. Here, we knocked down EFB1 and demonstrated that it is required for differentiation of hiPSCs into RGCs. We show that it acts in the early stages of OV morphogenesis, as a clearly defined neural retina precursor layer cannot form (Fig. 7c). Moreover, EBF1 acts upstream of RGC-specifying TFs BRN3A and ISL1, as their levels are downregulated upon EBF1 knockdown (Fig. 7a).

Of prominent interest are the TFs with particularly high overexpression in OVs, lower overexpression in differentiated RGCs and, at the same time, significant downregulation in RPE cells, as they are very likely to be important for the early stages of RGC differentiation from the OV RPCs. In our study, such an expression pattern was validated for the TF-encoding genes *DLX1*, *DLX2* and *INSM1* (Fig. 6a). The important role of *DLX1* and *DLX2* in RGC differentiation has been well characterized previously [38]. In a recent study it was found that mouse *Brn3b* expression is directly regulated by DLX1 and DLX2, thus emphasizing their role in the early stages of RGC development [39]. Given the fact that BRN3B is directly downstream of ATOH7, DLX1 and DLX2 may complement its function in initiation of RGC lineage specification. *INSM1* encodes a zinc finger TF with a repressive mode of action, which has been characterized as an important regulator of neuroendocrine system development [40]. It was shown to be required for retinal differentiation in zebrafish, where it acts upstream of *ath5/atoh7* and is negatively regulated by Notch signaling [41]. The latter observation is consistent with our data, as Notch inhibitor DAPT was used in our protocol of RGC differentiation. INSM1 has a repressive action on cell cycle and is believed to regulate the transition stage between late progenitors and nascent neurons [42], which is consistent with the observed expression pattern of drastic upregulation in OVs and reduced overexpression in mature RGCs (Figs. 5d and 6).

RPE cells had significantly less differentially expressed TFs, according to our highly stringent criteria, than OVs and RGCs (Fig. 5d). As expected, the most highly overexpressed TF in RPE cells was MITF, the master regulator of RPE differentiation [43]. Interestingly, among the TF genes most highly overexpressed in RPE cells, but not in OVs or RGCs, were two paralogs, *BHLHE41* and *BHLHE40* (Fig. 5d). These members of the bHLH family TFs were extensively characterized as important circadian rhythm regulators; however, they were also shown to be involved in various developmental processes [44]. Interestingly, BHLHE40 and BHLHE41 are known to negatively regulate TWIST1 [45], which is consistent with our data, as TWIST1 was in the top 10 of the most highly overexpressed TF-encoding genes in OVs and RGCs, but not in RPE cells.

The transcriptional activity of lineage-specific TFs is tightly linked to the chromatin state of the genome. The pluripotent stem cells undergo genome-wide chromatin remodeling during differentiation. In general, the genomes of ESCs/iPSCs are characterized by a fairly open chromatin state that is permissive for global transcription; at the same time, lineage-specific regulatory genes are maintained in a transcriptionally silenced chromatin configuration [46, 47]. In the process of differentiation, chromatin undergoes global change into a more compact, heterochromatic configuration, whereas lineage-specific regulators are transcriptionally activated [46, 47]. The chromatin state is regulated by various mechanisms that include DNA modifications (e.g., cytosine methylation), post-translational modifications of histones (e.g., methylation, acetylation and ubiquitination) and ATP-dependent remodeling of chromatin. In our study, we investigated differential expression of components of PcG (including PRC1 and PRC2) and TrxG complexes (including COMPASS/MLL and SWI/SNF), two groups of chromatin-modifying complexes that have opposing effects on transcriptional activity: the former repressive, the latter activating. In contrast to TFs, the patterns of regulation of components of epigenetic complexes were more similar between RGCs and RPE cells, which may reflect the less specific but more general requirement for epigenetic complexes to create the correct chromatin environment for the action of lineage-specific TFs. Most of the components of COMPASS/MLL complexes, that perform activating chromatin methylation H3K4-me3, were downregulated upon differentiation, which is consistent with a more permissive chromatin environment in hiPSCs.

The heterogeneous composition of various chromatin modifier complexes may define their specificity and therefore may lead to different lineage specification. For example, PCGF homologs, CBX proteins and RYBP define functionally distinct PRC1 Family Complexes [48]. Six different classes of PRC1 complexes are defined by the identity of their six PCGF subunits, PRC1.1–PRC1.6 [48]. Here, we show that the most overexpressed PCGF subunit was PCGF5, thus making the PRC1.5 complex the most prevalent variant in retinal lineages, which is consistent with its association with neuronal function [48]. At the same time, PCGF6 was downregulated in differentiated retinal lineages, which is consistent with its role in embryonic stem cell maintenance [49].

Interestingly, among the components of SWI/SNF chromatin remodeling complexes, ACTL6B demonstrated a significantly contrasted expression pattern: it was significantly downregulated in RPE cells but, on the other hand, it was highly upregulated in the OV/RGC lineage. Given the fact that ACTL6B is a component of the neuronal-specific SWI/SNF complex (nBAF) that participates in postmitotic neural development and dendritic outgrowth [50], it may play a similar role in RGC development.

## Conclusions

To summarize, we identified novel potentially important intrinsic regulators of RGC and RPE cell lineage specification in a process of differentiation from hiPSCs. In addition, we identified intrinsic regulator biomarker signatures of these two retinal cell types that can be applied with high confidence to confirm the cell lineage identities. We show that EBF1 is required for differentiation of hiPSCs into RGCs via the OV stage, and it acts upstream of the crucial regulators of RGC specification BRN3A and ISL1. This study identified a number of novel potential crucial regulators of retinal lineage specification for future characterization.

## Additional file

Additional file 1: Primers. (XLSX 11 kb)

## References

[1] Takahashi K, Yamanaka S (2006). Induction of pluripotent stem cells from mouse embryonic and adult fibroblast cultures by defined factors. Cell.

[2] Takahashi K, Tanabe K, Ohnuki M, Narita M, Ichisaka T, Tomoda K, Yamanaka S (2007). Induction of pluripotent stem cells from adult human fibroblasts by defined factors. Cell.

[3] Tang Z, Zhang Y, Wang Y, Zhang D, Shen B, Luo M, Gu P (2017). Progress of stem/progenitor cell-based therapy for retinal degeneration. J Transl Med.

[4] Wright LS, Phillips MJ, Pinilla I, Hei D, Gamm DM (2014). Induced pluripotent stem cells as custom therapeutics for retinal repair: progress and rationale. Exp Eye Res.

[5] Gill KP, Hewitt AW, Davidson KC, Pébay A, Wong RC (2014). Methods of retinal ganglion cell differentiation from pluripotent stem cells. Transl Vis Sci Technol.

[6] Klimanskaya I, Hipp J, Rezai KA, West M, Atala A, Lanza R (2004). Derivation and comparative assessment of retinal pigment epithelium from human embryonic stem cells using transcriptomics. Cloning Stem Cells.

[7] Buchholz DE, Hikita ST, Rowland TJ, Friedrich AM, Hinman CR, Johnson LV, Clegg DO (2009). Derivation of functional retinal pigmented epithelium from induced pluripotent stem cells. Stem Cells.

[8] Osakada F, Ikeda H, Mandai M, Wataya T, Watanabe K, Yoshimura N, Akaike A, Sasai Y, Takahashi M (2008). Toward the generation of rod and cone photoreceptors from mouse, monkey and human embryonic stem cells. Nat Biotechnol.

[9] Osakada F, Jin ZB, Hirami Y, Ikeda H, Danjyo T, Watanabe K, Sasai Y, Takahashi M (2009). In vitro differentiation of retinal cells from human pluripotent stem cells by small-molecule induction. J Cell Sci.

[10] Idelson M, Alper R, Obolensky A, Ben-Shushan E, Hemo I, Yachimovich-Cohen N, Khaner H, Smith Y, Wiser O, Gropp M, Cohen MA, Even-Ram S, Berman-Zaken Y, Matzrafi L, Rechavi G, Banin E, Reubinoff B (2009). Directed differentiation of human embryonic stem cells into functional retinal pigment epithelium cells. Cell Stem Cell.

[11] Zahabi A, Shahbazi E, Ahmadieh H, Hassani SN, Totonchi M, Taei A, Masoudi N, Ebrahimi M, Aghdami N, Seifinejad A, Mehrnejad F, Daftarian N, Salekdeh GH, Baharvand H (2012). A new efficient protocol for directed differentiation of retinal pigmented epithelial cells from normal and retinal disease induced pluripotent stem cells. Stem Cells Dev.

[12] Buchholz DE, Pennington BO, Croze RH, Hinman CR, Coffey PJ, Clegg DO (2013). Rapid and efficient directed differentiation of human pluripotent stem cells into retinal pigmented epithelium. Stem Cells Transl Med.

[13] Schwartz SD, Regillo CD, Lam BL, Eliott D, Rosenfeld PJ, Gregori NZ, Hubschman JP, Davis JL, Heilwell G, Spirn M, Maguire J, Gay R, Bateman J, Ostrick RM, Morris D, Vincent M, Anglade E, Del Priore LV, Lanza R (2015). Human embryonic stem cell-derived retinal pigment epithelium in patients with age-related macular degeneration and Stargardt's macular dystrophy: follow-up of two open-label phase 1/2 studies. Lancet.

[14] Zhao C, Wang Q, Temple S (2017). Stem cell therapies for retinal diseases: recapitulating development to replace degenerated cells. Development.

[15] Riazifar H, Jia Y, Chen J, Lynch G, Huang T (2014). Chemically induced specification of retinal ganglion cells from human embryonic and induced pluripotent stem cells. Stem Cells Transl Med.

[16] Nguyen M, Arnheiter H (2000). Signaling and transcriptional regulation in early mammalian eye development: a link between FGF and MITF. Development.

[17] Rowan S, Chen CM, Young TL, Fisher DE, Cepko CL (2004). Transdifferentiation of the retina into pigmented cells in ocular retardation mice defines a new function of the homeodomain gene Chx10. Development.

[18] Horsford DJ, Nguyen MT, Sellar GC, Kothary R, Arnheiter H, McInnes RR (2005). Chx10 repression of Mitf is required for the maintenance of mammalian neuroretinal identity. Development.

[19] Yang Z, Ding K, Pan L, Deng M, Gan L (2003). Math5 determines the competence state of retinal ganglion cell progenitors. Dev Biol.

[20] Wang SW, Kim BS, Ding K, Wang H, Sun D, Johnson RL, Klein WH, Gan L (2001). Requirement for math5 in the development of retinal ganglion cells. Genes Dev.

[21] Brown NL, Patel S, Brzezinski J, Glaser T (2001). Math5 is required for retinal ganglion cell and optic nerve formation. Development.

[22] Mu X, Fu X, Beremand PD, Thomas TL, Klein WH (2008). Gene regulation logic in retinal ganglion cell development: Isl1 defines a critical branch distinct from but overlapping with Pou4f2. Proc Natl Acad Sci U S A.

[23] Sun Y, Dykes IM, Liang X, Eng SR, Evans SM, Turner EE (2008). A central role for Islet1 in sensory neuron development linking sensory and spinal gene regulatory programs. Nat Neurosci.

[24] Sapkota D, Chintala H, Wu F, Fliesler SJ, Hu Z, Mu X (2014). Onecut1 and Onecut2 redundantly regulate early retinal cell fates during development. Proc Natl Acad Sci U S A.

[25] Mao CA, Kiyama T, Pan P, Furuta Y, Hadjantonakis AK, Klein WH (2008). Eomesodermin, a target gene of Pou4f2, is required for retinal ganglion cell and optic nerve development in the mouse. Development.

[26] Shi M, Kumar SR, Motajo O, Kretschmer F, Mu X, Badea TC (2013). Genetic interactions between Brn3 transcription factors in retinal ganglion cell type specification. PLoS One.

[27] Sajgo S, Ghinia MG, Brooks M, Kretschmer F, Chuang K, Hiriyanna S, Wu Z, Popescu O, Badea TC (2017). Molecular codes for cell type specification in Brn3 retinal ganglion cells. Proc Natl Acad Sci U S A.

[28] Gautier L, Cope L, Bolstad BM, Irizarry RA (2004). affy—analysis of Affymetrix GeneChip data at the probe level. Bioinformatics.

[29] Ritchie ME, Phipson B, Wu D, Hu Y, Law CW, Shi W, Smyth GK (2015). limma powers differential expression analyses for RNA-sequencing and microarray studies. Nucleic Acids Res.

[30] Mi H, Huang X, Muruganujan A, Tang H, Mills C, Kang D, Thomas PD (2017). PANTHER version 11: expanded annotation data from Gene Ontology and Reactome pathways, and data analysis tool enhancements. Nucleic Acids Res.

[31] Poynter ST, Kadoch C (2016). Polycomb and trithorax opposition in development and disease. Wiley Interdiscip Rev Dev Biol.

[32] Medvedeva YA, Lennartsson A, Ehsani R, Kulakovskiy IV, Vorontsov IE, Panahandeh P, Khimulya G, Kasukawa T, Drabløs F, FANTOM Consortium (2015). EpiFactors: a comprehensive database of human epigenetic factors and complexes. Database (Oxford).

[33] Heavner W, Pevny L (2012). Eye development and retinogenesis. Cold Spring Harb Perspect Biol.

[34] Halbleib JM, Nelson WJ (2006). Cadherins in development: cell adhesion, sorting, and tissue morphogenesis. Genes Dev.

[35] Zhang HM, Chen H, Liu W, Liu H, Gong J, Wang H, Guo AY (2012). AnimalTFDB: a comprehensive animal transcription factor database. Nucleic Acids Res.

[36] Trimarchi JM, Stadler MB, Roska B, Billings N, Sun B, Bartch B, Cepko CL (2007). Molecular heterogeneity of developing retinal ganglion and amacrine cells revealed through single cell gene expression profiling. J Comp Neurol.

[37] Jin K, Jiang H, Mo Z, Xiang M (2010). Early B-cell factors are required for specifying multiple retinal cell types and subtypes from postmitotic precursors. J Neurosci.

[38] de Melo J, Du G, Fonseca M, Gillespie LA, Turk WJ, Rubenstein JL, Eisenstat DD (2005). Dlx1 and Dlx2 function is necessary for terminal differentiation and survival of late-born retinal ganglion cells in the developing mouse retina. Development.

[39] Zhang Q, Zagozewski J, Cheng S, Dixit R, Zhang S, de Melo J, Mu X, Klein WH, Brown NL, Wigle JT, Schuurmans C, Eisenstat DD (2017). Regulation of Brn3b by DLX1 and DLX2 is required for retinal ganglion cell differentiation in the vertebrate retina. Development.

[40] Lan MS, Breslin MB (2009). Structure, expression, and biological function of INSM1 transcription factor in neuroendocrine differentiation. FASEB J.

[41] Forbes-Osborne MA, Wilson SG, Morris AC (2013). Insulinoma-associated 1a (Insm1a) is required for photoreceptor differentiation in the zebrafish retina. Dev Biol.

[42] Duggan A, Madathany T, de Castro SC, Gerrelli D, Guddati K, García-Añoveros J (2008). Transient expression of the conserved zinc finger gene INSM1 in progenitors and nascent neurons throughout embryonic and adult neurogenesis. J Comp Neurol.

[43] Tachibana M (2000). MITF: a stream flowing for pigment cells. Pigment Cell Res.

[44] Ow JR, Tan YH, Jin Y, Bahirvani AG, Taneja R (2014). Stra13 and Sharp-1, the non-grouchy regulators of development and disease. Curr Top Dev Biol.

[45] Suzuki M, Sato F, Bhawal UK (2014). The basic helix-loop-helix (bHLH) transcription factor DEC2 negatively regulates Twist1 through an E-box element. Biochem Biophys Res Commun.

[46] Chen T, Dent SY (2014). Chromatin modifiers and remodellers: regulators of cellular differentiation. Nat Rev Genet.

[47] Festuccia N, Gonzalez I, Navarro P (2017). The epigenetic paradox of pluripotent ES cells. J Mol Biol.

[48] Gao Z, Zhang J, Bonasio R, Strino F, Sawai A, Parisi F, Kluger Y, Reinberg D (2012). PCGF homologs, CBX proteins, and RYBP define functionally distinct PRC1 family complexes. Mol Cell.

[49] Zhao W, Tong H, Huang Y, Yan Y, Teng H, Xia Y, Jiang Q, Qin J (2017). Essential role for polycomb group protein Pcgf6 in embryonic stem cell maintenance and a noncanonical polycomb repressive complex 1 (PRC1) integrity. J Biol Chem.

[50] Sokpor G, Xie Y, Rosenbusch J, Tuoc T (2017). Chromatin remodeling BAF (SWI/SNF) complexes in neural development and disorders. Front Mol Neurosci.

